# Bibliometric analysis of imaging and treatment strategies for severe tricuspid regurgitation from 2015 to 2023

**DOI:** 10.3389/fcvm.2024.1444466

**Published:** 2024-10-29

**Authors:** Johannes Schlegl, Marwin Bannehr, Tanja Kücken, Paulina Jankowska, Michael Neuss, Michael Lichtenauer, Anja Haase-Fielitz, Christian Butter, Christoph Edlinger

**Affiliations:** ^1^ Department of Cardiology, University Hospital Heart Center Brandenburg, Brandenburg Medical School (MHB) Theodor Fontane, Neuruppin, Germany; ^2^ Faculty of Health Sciences Brandenburg, Brandenburg Medical School Theodor Fontane, Neuruppin, Germany; ^3^ Department of Cardiology, Clinic of Internal Medicine II, Paracelsus Medical University of Salzburg, Salzburg, Austria; ^4^ Institute of Social Medicine and Health System Research, Otto von Guericke University Magdeburg, Magdeburg, Germany

**Keywords:** tricuspid regurgitation, bibliometric analysis, edge-to-edge-treatment, heat maps, tricuspid valve

## Abstract

**Background:**

Severe tricuspid regurgitation is a progressive disease with an unfavourable prognosis. In recent years there have been extraordinary gains in knowledge through both clinical and basic scientific work. We performed a bibliometric analysis on tricuspid regurgitation with a focus on imaging techniques and treatment approaches and to identify scientific milestones and emerging research trends.

**Methods:**

Publications, published between 2015 and 2023 were identified. Study characteristics, impact factors and countries of origin studies were recorded. Heat maps were created to visualise data and to identify leading centers. Most frequently cited publications were recognised as milestones.

**Results:**

We screened 3,519 studies. 368 studies were included, of which 326 were clinical studies. Clinical studies were further subdivided into interventional (*n* = 138), surgical (*n* = 115) or studies on imaging modalities (*n* = 74). We detected an enormous increase in scientific output worldwide, especially in imaging and interventional studies. The United States, Germany and Poland were identified as leading countries in imaging, interventions and preclinical studies respectively.

**Conclusions:**

Our study reflects the global gain in knowledge over the last 9 years. We were able to identify an annually rising number of interventional studies. Imaging studies have also seen a rapid increase, especially since 2020. In recent years, we monitored a decline in surgical studies.

## 1 Introduction

Tricuspid regurgitation (TR) is a multifaceted and complex disease with a poor prognosis if left untreated (1, 2). The increased mortality rate is mainly due to progressive right heart failure with consecutive impairment of other organ systems such as the kidneys and liver (3, 4). Although immense progress has been made in the diagnosis and treatment of valvular heart disease in recent decades, TR has long led a sort of shadowy existence and for this reason has also been frequently referred to as “the forgotten heart valve” (5–7).

This can be explained by the fact that, on the one hand, TR was considered less or not at all relevant compared to other pathologies such as aortic stenosis, mitral regurgitation and aortic regurgitation and, on the other hand, the surgical results (except in the case of simultaneous treatment of left ventricular failure) did not lead to an improvement in outcome (8, 9). Favoured by the widespread availability of improved imaging techniques, knowledge of TR has also increased in recent years and interventional treatment of the disease is becoming increasingly common in clinical practice (10, 11). Initially, it was recognised that coexisting TR may have prognostic relevance in cardiac surgery, and in many cases concomitant repair may be required (12, 13). In addition, the increase in im-aging techniques has led to a significant gain in knowledge about prognosis and mortality (14). Hahn et al. have developed a new echocardiographic classification of the severity of TR, which allows better classification of patients (15, 16). In recent years, there has been a continuous development of interventional treatment, with imaging studies also gaining in importance alongside “edge-to-edge” procedures, which largely correspond to the better-researched mitral valve interventions (17, 18). For patients in whom an edge-to-edge procedure is not possible due to anatomical conditions, a wide range of devices has been developed for complete interventional tricuspid valve replacement as well as for heterotopic minimally invasive tricuspid valve replacement (19–21).

Nevertheless, there are currently gaps in knowledge such as in the selection of patients and the timing of interventions. It is also currently not yet been fully understood what significance concomitant diseases of other organ systems such as the kidneys and liver might have for the prognosis. There are also no defined cut-off values as to when the disease has progressed too far, a sustainable treatment success is unlikely and a conservative procedure in terms of a palliative treatment approach should possibly be sought.

The aim of this bibliometric study was to analyze the quantity of research activity, the quality and to identify trends in the field of TR. The most frequently cited publications were defined as milestones and shall be presented separately for the three sections surgical, interventional and imaging.

## 2 Materials and methods

All data from publications on TR were extracted from PubMed by the U.S. National Library of Medicine. The studies found to be suitable were transferred to a Microsoft Excel database.

### 2.1 Search strategy

First, we defined search terms to include as many publications on TR from the period 2015–2023 as possible using a PubMed search. Screening of the literature was performed by three colleagues working independently of each other. Data was collected between May 1st, 2023 and January 31st, 2024. The combined search terms were “interventional tricuspid repair” OR “T-TEER” OR “treatment of severe tricuspid regurgitation”. The search terms identified 3,519 publications, which were manually screened in a further step to clarify whether they met the inclusion criteria listed below. Finally, 368 publications were included in the analysis.

### 2.2 Inclusion/exclusion criteria

Inclusion criteria comprised preclinical and original clinical studies. Clinical studies were further subdivided into “imaging studies”, “cardiac surgery studies” and “interventional-cardiological” studies. Excluded were reviews, meta-analyses, case reports and paediatric studies on congenital valvular heart diseases.

### 2.3 Heat maps

Both the quantity (based on overall output) and the quality (based on the impact factors) were surveyed and documented. In this study, we created heat maps to illustrate global development, the distribution of leading countries and scientific quality. The country of origin of the corresponding author was always used for the classification in the heat maps. This methodology, in which leading centres are depicted with increasing red colouring, has already been used in epidemiological or medical economic studies (22, 23). For visualisation, the data was processed using Someka Excel Solutions (Someka, Izmir, Turkey), a commercially available software that can be used to create “heat maps”. With this method, the scientific output can be reflected by showing particularly leading countries in dark red, while less relevant countries are shown in a lighter colour. Accordingly, countries without scientific output in the analysed period are shown in white. Leading research nations were identified based on the following criteria: Number of studies, impact factor of the studies and number of citations of the studies. The impact factors of the respective publication year were obtained for all identified publications from 2015 to 2023 from www.bioxbio.com, a publicly accessible homepage. Impact factors from the year of publication of the respective paper were used throughout the study.

### 2.4 Milestones

The number of citations was recorded and documented after each individual check using the PubMed database. The most frequently cited publications for each year were recorded for the imaging, surgical and interventional sub-areas and visualised as “milestones” on a timeline. We created one timeline for each clinical category to monitor the entire spectrum of clinical gain in knowledge in this respective topic.

## 3 Results

### 3.1 Chronological map of the literature

The number of research articles on tricuspid regurgitation initially trended upwards from 2015. There was a cesura in 2019, whereby an even more significant increase was evident from 2020 and especially from 2021.

### 3.2 Analysis of study focus and quality of the literature

**Figure 1:**
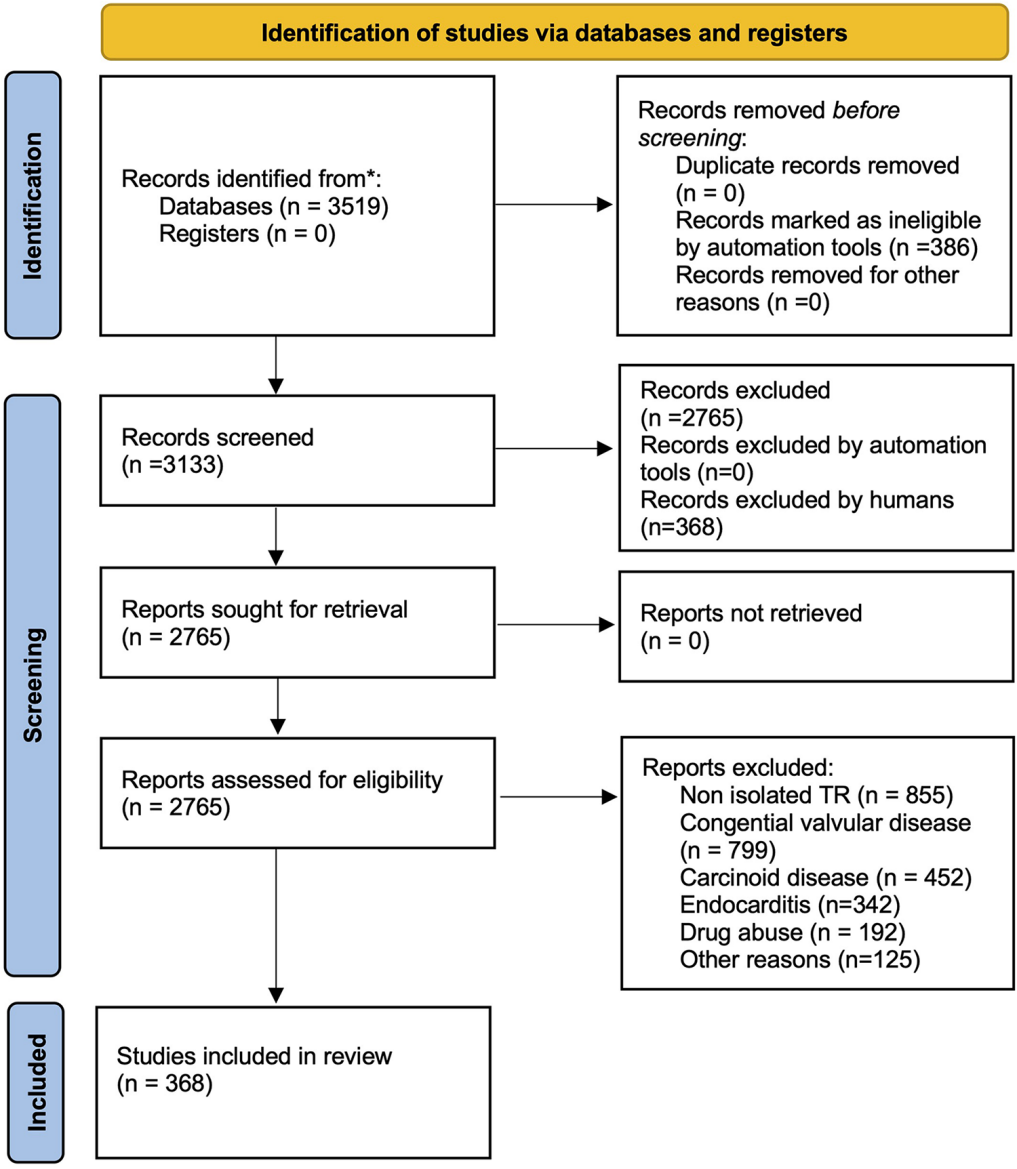
Flow chart on search strategy.

**Figure 2:**
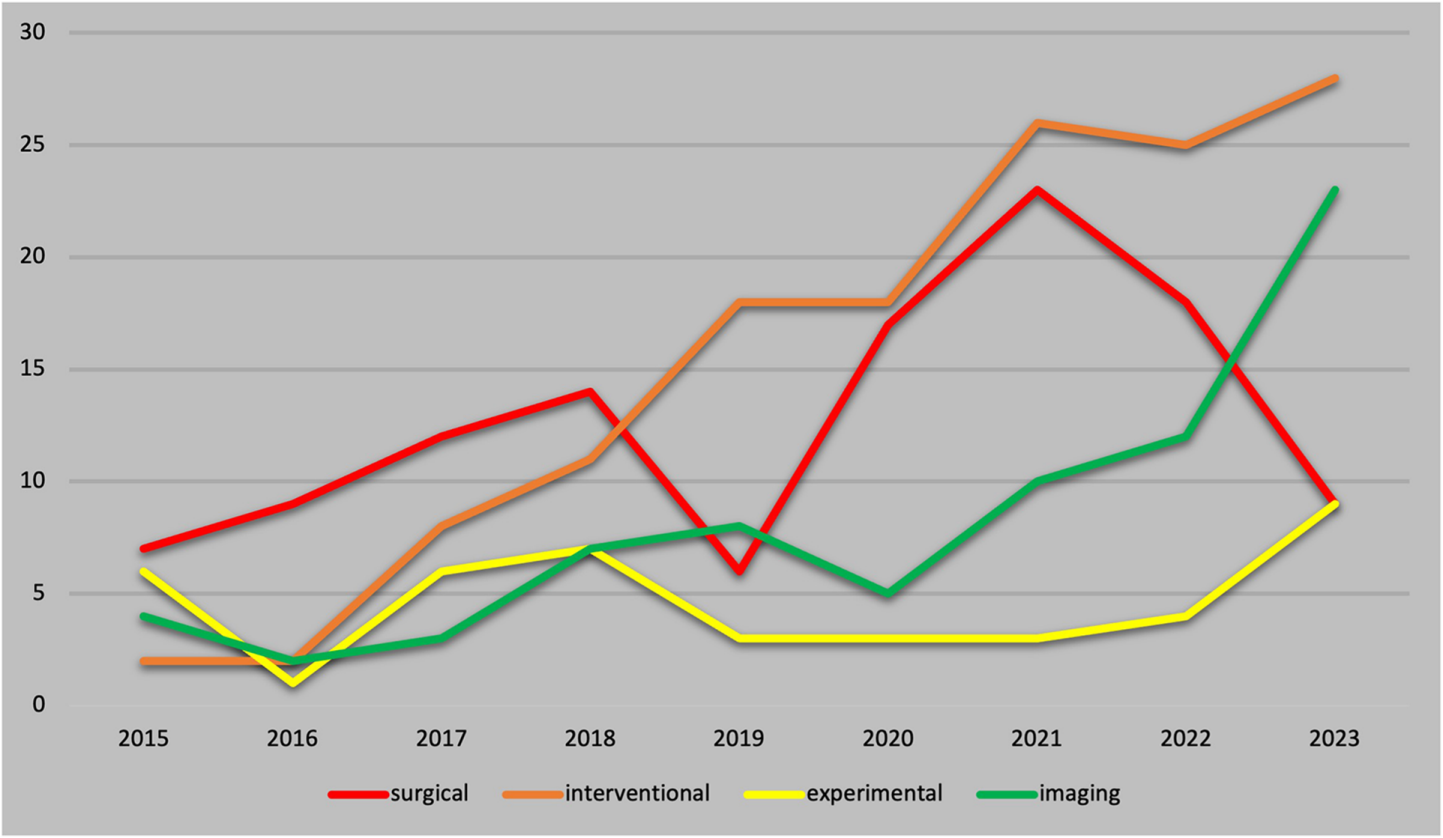
Annual number of publications from 2015 to 2023.

327 of the publication identified were categorized as clinical studies and 42 as pre-clinical studies. An overview of the selection process and the categorization can be found in Figure 1. Clinical studies were further categorized as interventional cardiology studies (*n* = 138), surgical (*n* = 115) and imaging studies (*n* = 74). Figure 2 shows the development within the individual sub-areas: “surgical”, “interventional”, “imaging” and “preclinical” over the years. The number of preclinical studies per year has not increased over the 9 years examined and remains at a moderate level. No clear numerical trend can be discerned here. The number of surgical publications initially showed a continuous annual increase, which reached its maximum in 2021. Since then, there has been an abrupt and rapid decline of over 60%. The number of surgical publications has thus fallen back to the 2016 level within 2 years. In 2023, a similar number of surgical studies were published as preclinical studies. In the last 5 years, interventional and imaging studies have experienced stable growth. Since 2018, interventional studies have accounted for the most publications per year. The number of imaging studies has increased, especially since 2020, with a further increase of over 92% in 2023. All categories show a caesura within the years of the corona pandemic.

Preclinical studies had a cumulative impact factor of 144.5, while the clinical studies had a cumulative impact factor of 2,794.9. This resulted in a mean impact factor of 3.5 for preclinical studies and of 5.1 for clinical studies. Publications between 2015 and 2019 were cited with higher frequency, and the most cited articles were published in 2016 so far. The mean number of citations per publication over the entire period of analysis was 5.0, with the most cited countries being the US and Germany.

### 3.3 National distribution of the literature

**Figure 3:**
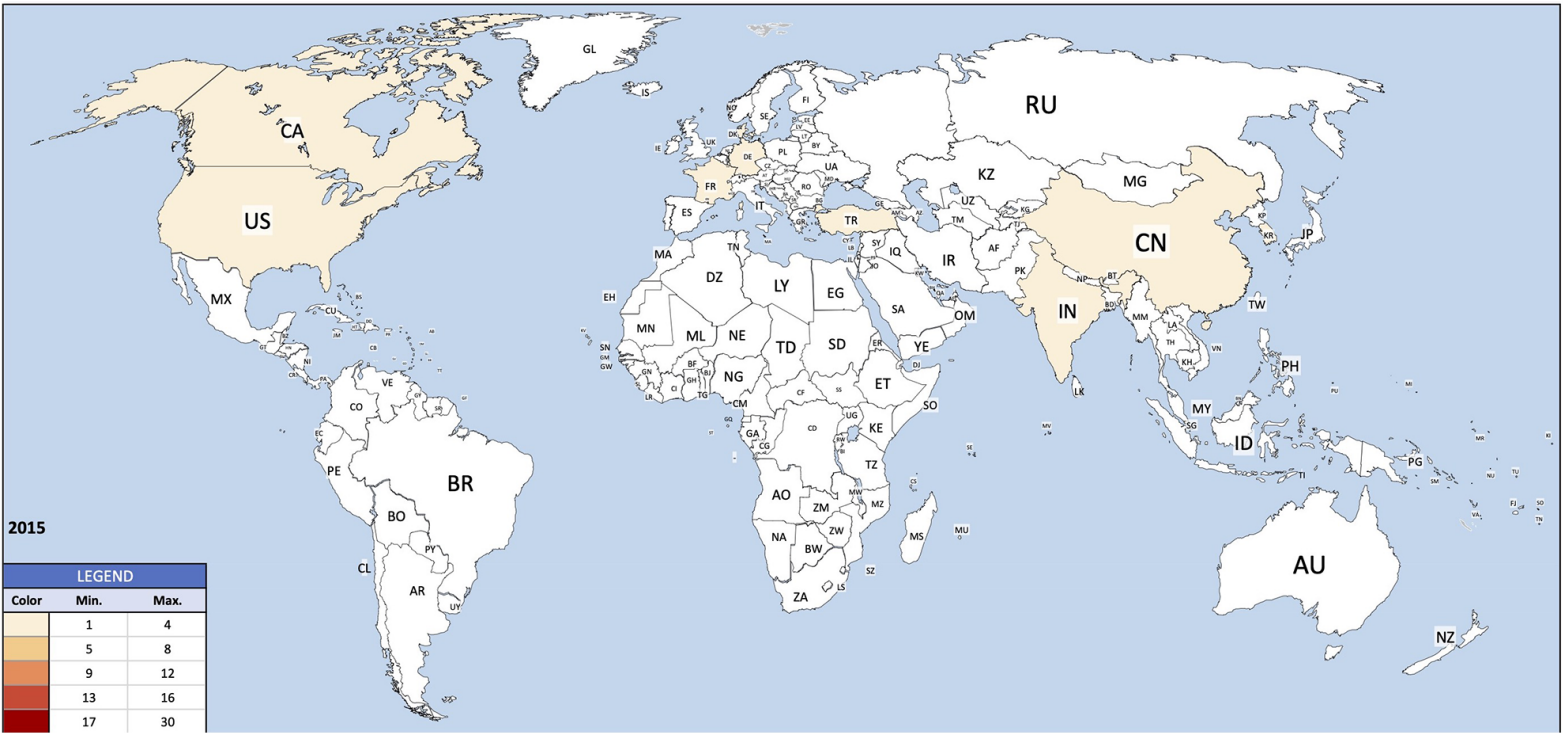
Heat map 2015.

**Figure 4:**
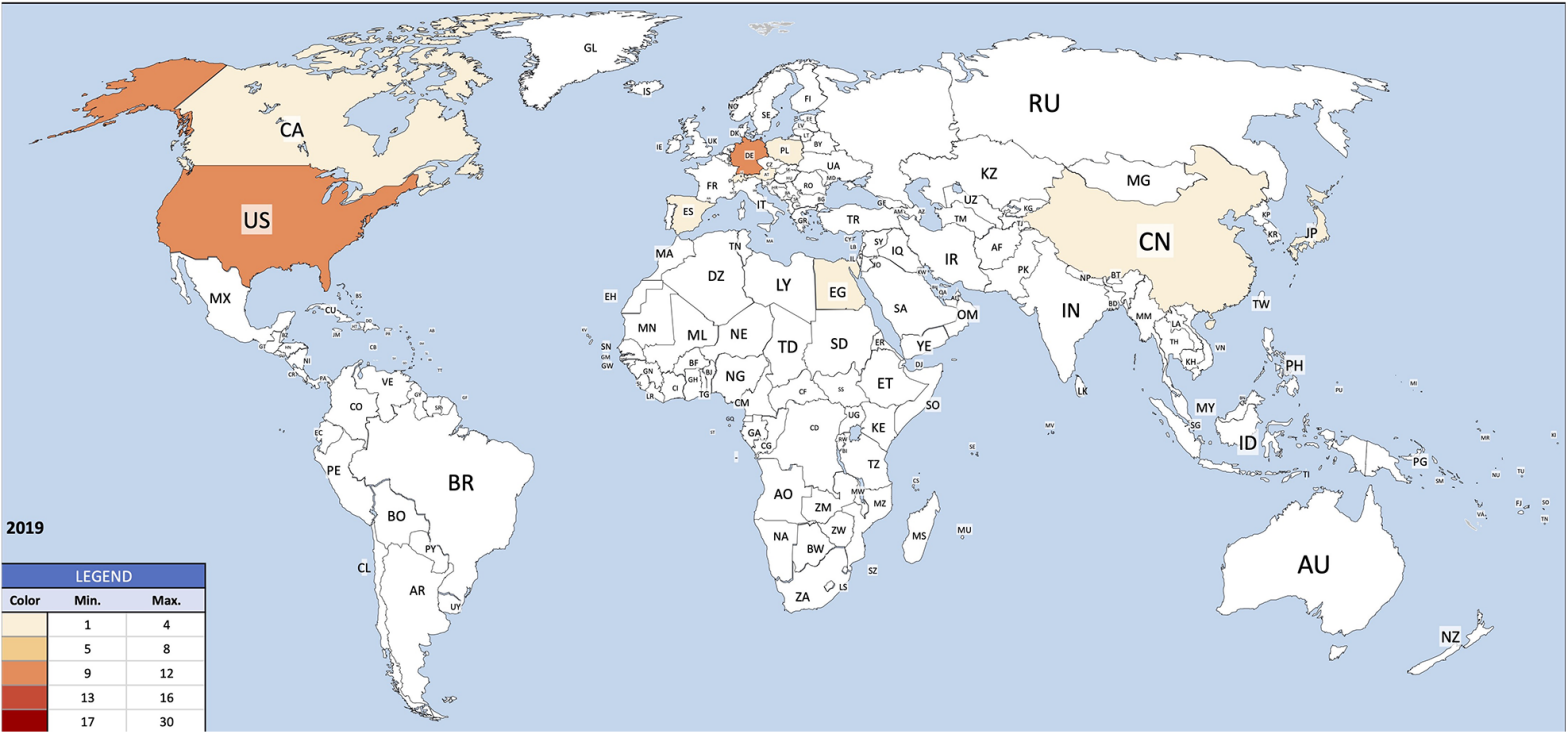
Heat map 2019.

**Figure 5:**
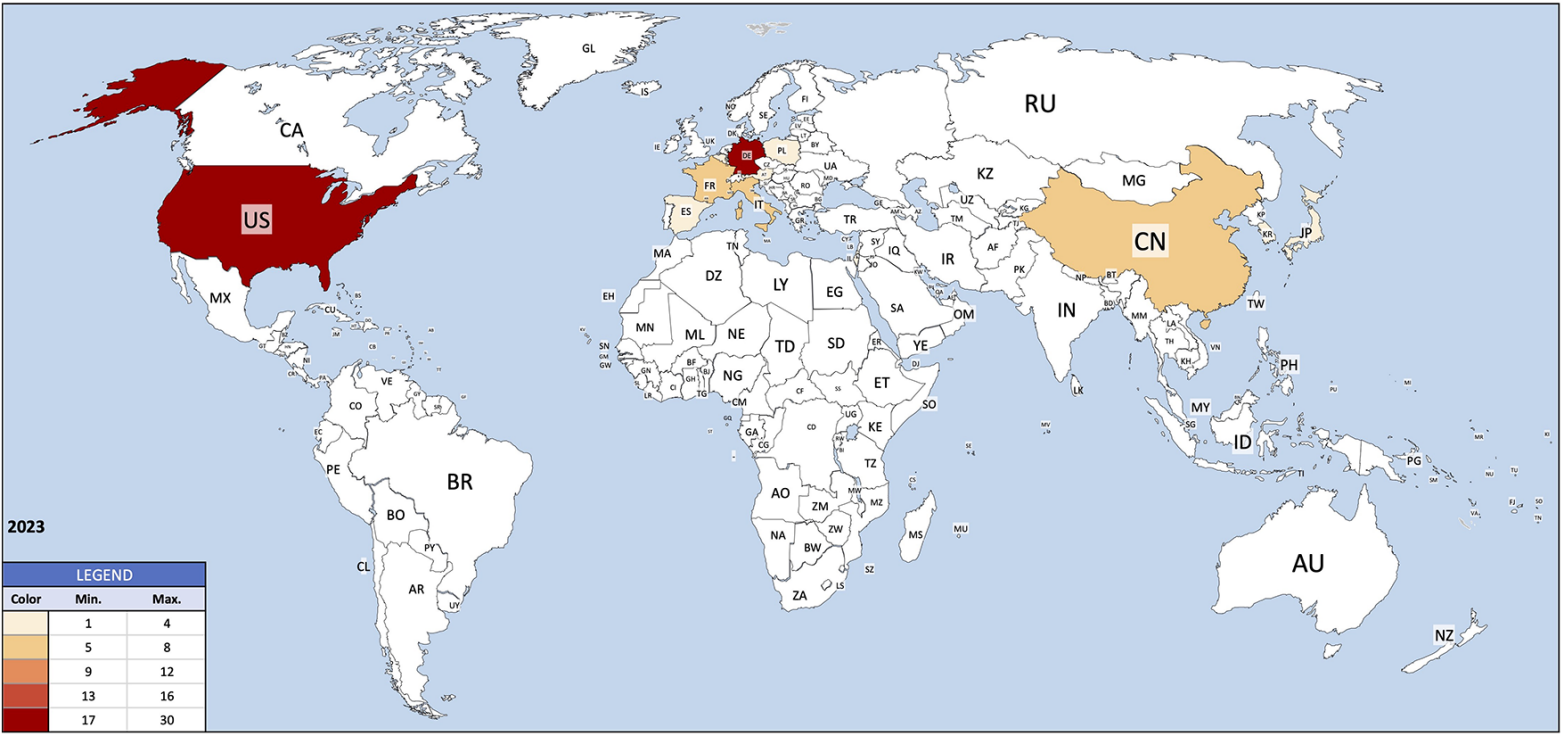
Heat map 2023.

**Figure 6:**
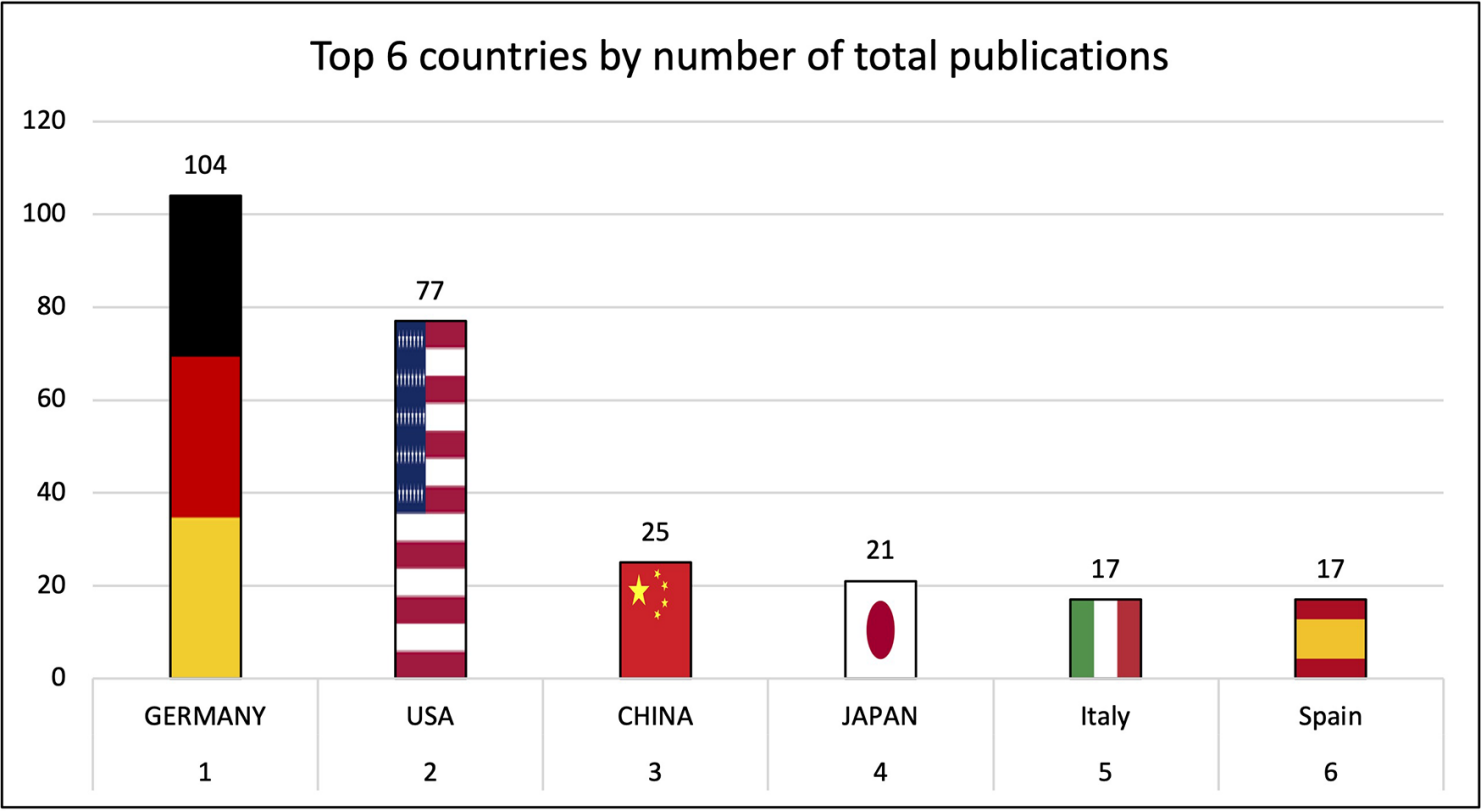
Leading countries by number of total publications.

**Figure 7:**
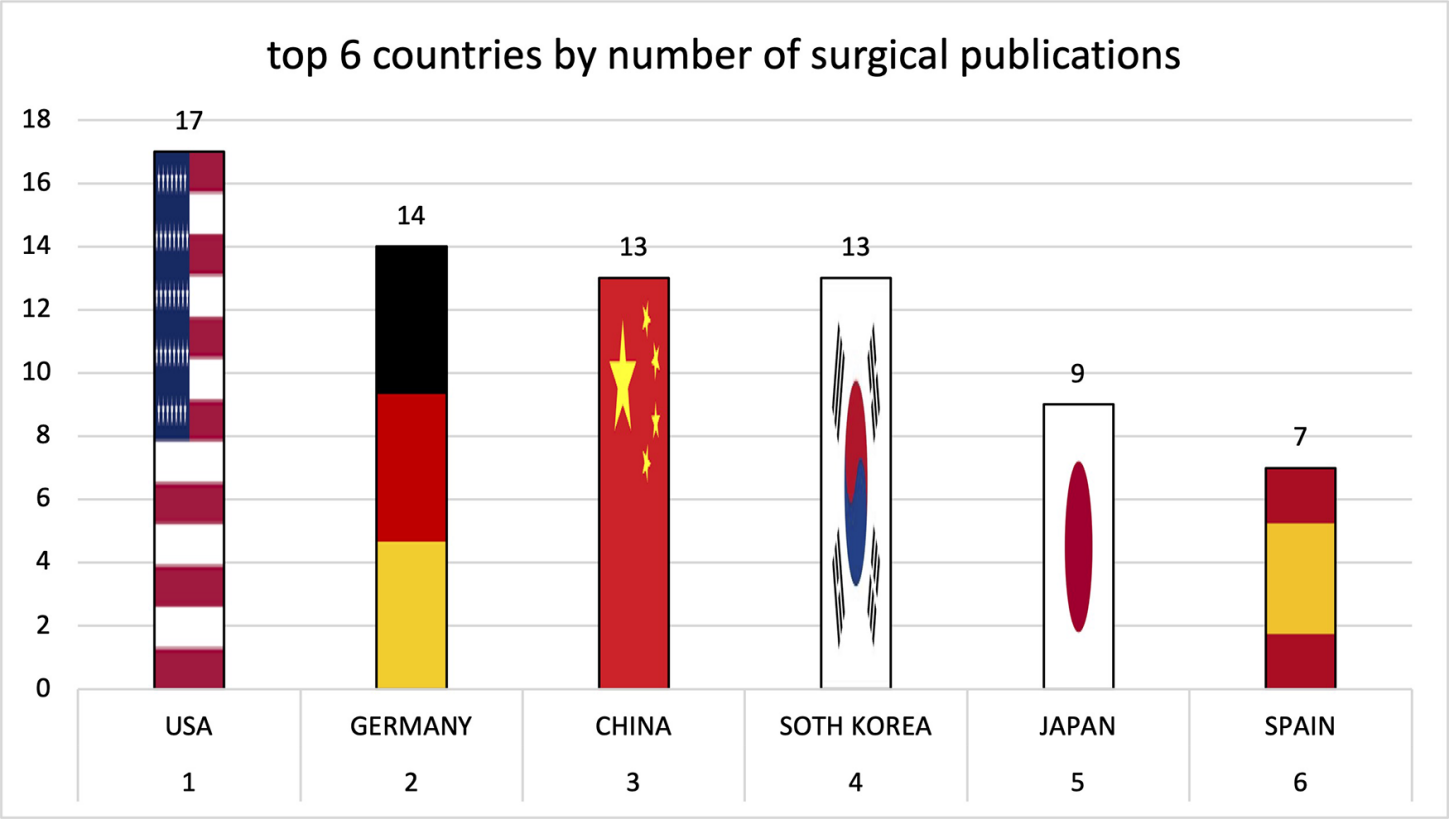
Leading countries by number of surgical publications.

**Figure 8:**
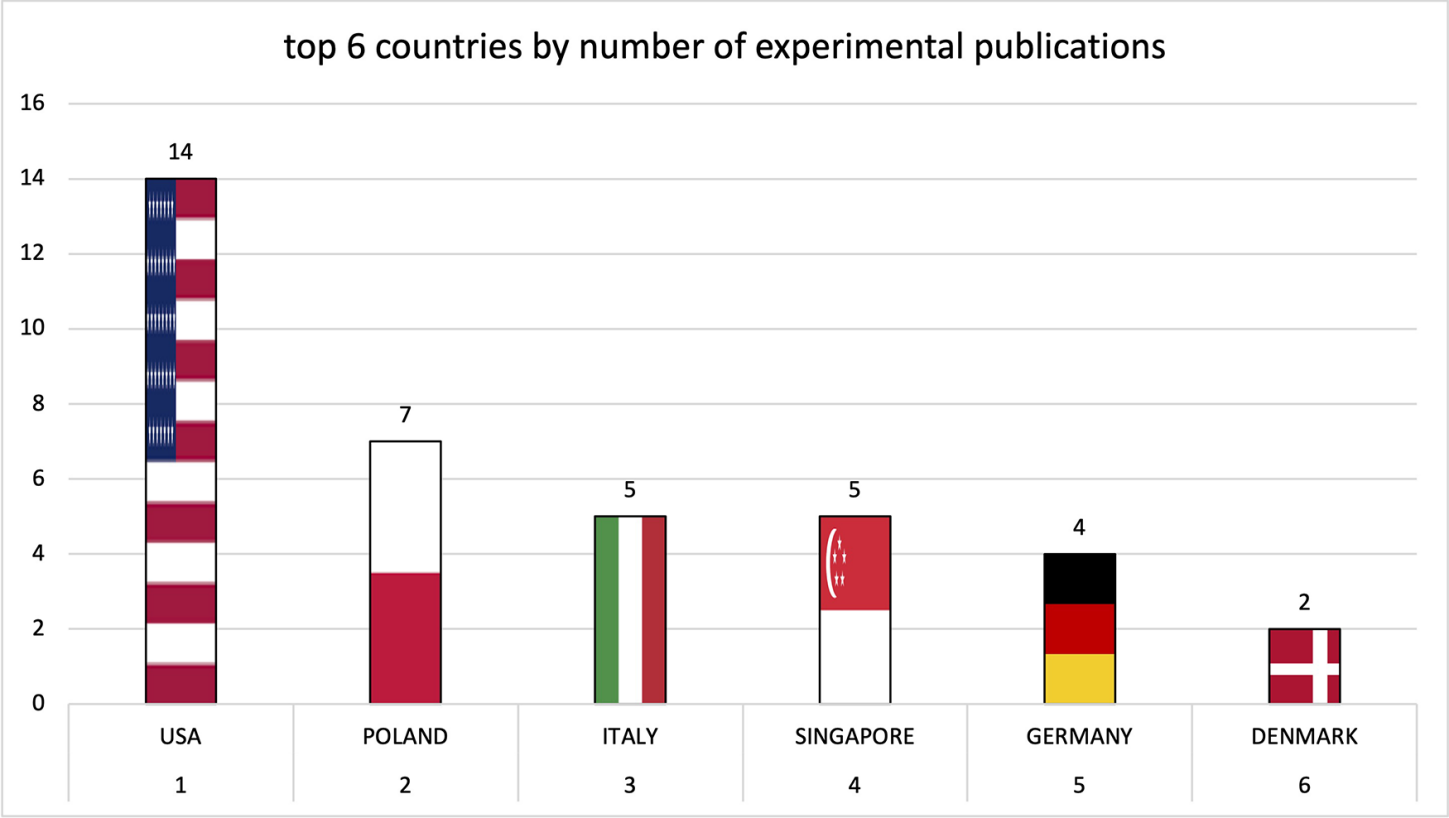
Leading countries by number of interventional publications.

**Figure 9:**
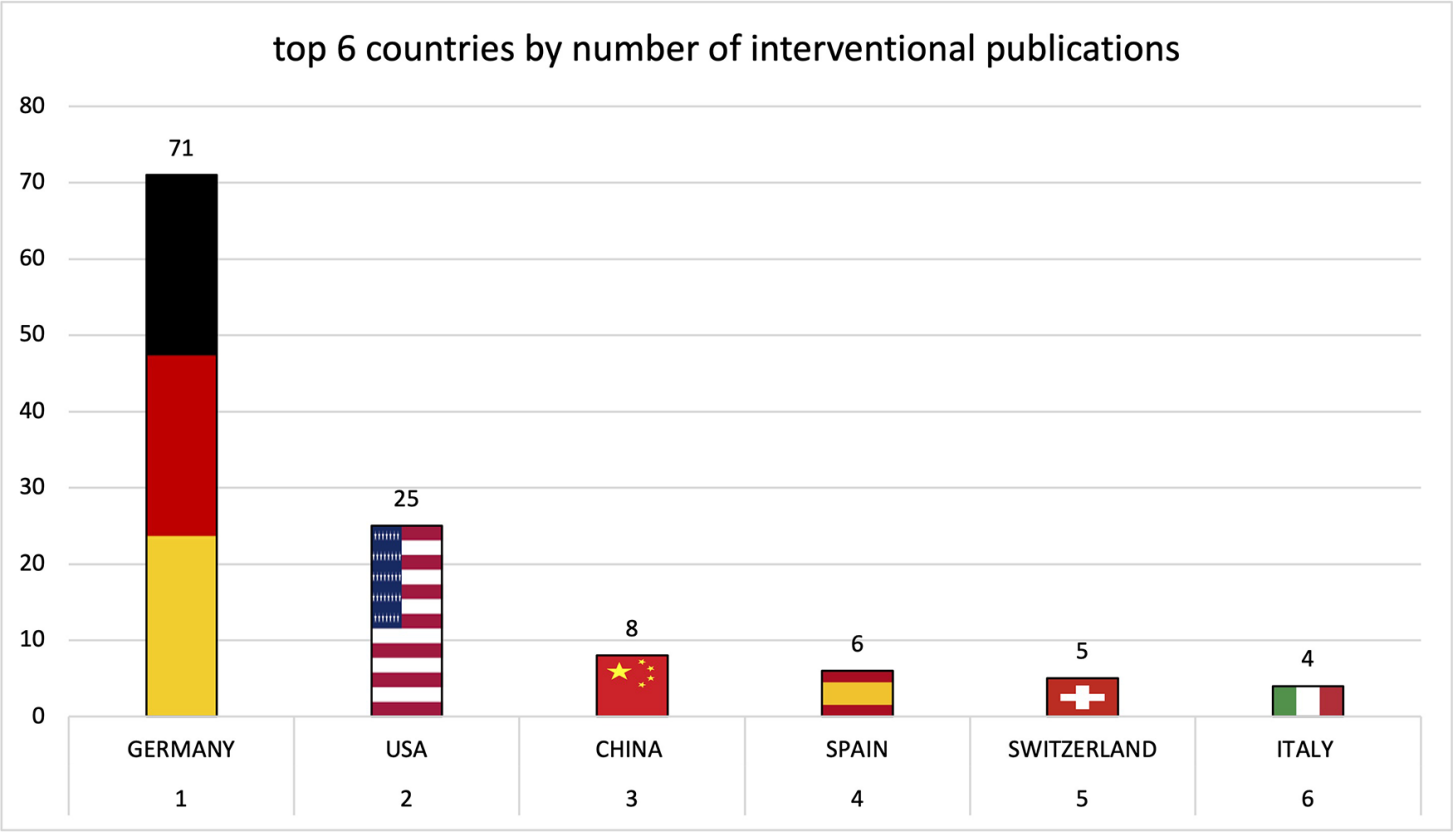
Leading countries by number of imaging publications.

**Figure 10:**
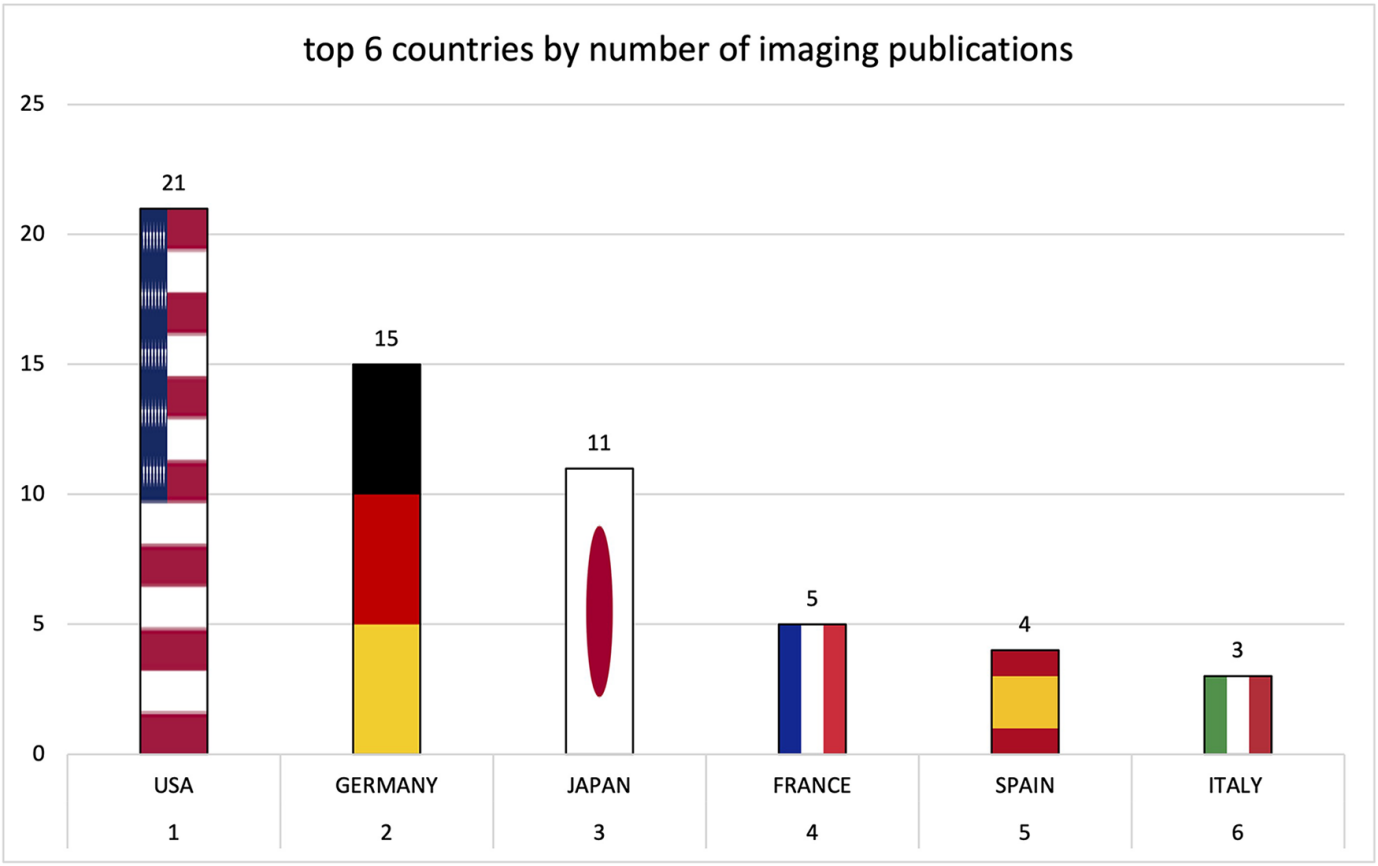
Leading countries by number of preclinical publications.

Institutions from Germany and the United States published the most articles during this 9-year period, accounting for 28.3% and 20.9% of the total number of articles, respectively. The analysis of the heat maps showed that initially the USA, Germany and China were pioneers in this particular field with Canada and France following close by. Figure 3 illustrates leading countries in global development during 2015. Furthermore as examples for the 9 year observation period Figure 4 (2019) and Figure 5 (2023) show the process of publication numbers worldwide. Heat maps for all analysed years from 2015 to 2023 can be found in the Supplementary Material. The number of publications per country in the respective research categories was also visualized separately in Figures 6–10.

Over the years, the USA and Germany have established themselves as the leading nations in the field of research on tricuspid regurgitation, with the USA focusing on im-aging and Germany leading in interventional studies. Comparatively smaller countries such as Poland also played a leading role in preclinical studies. The graphs of the most productive countries in each subcategory can be found in the Supplementary Material.

The clinical studies had a cumulative number of 3,141 citations with a median of 5. The surgical studies were cited a total of 558 times in 9 years with a median of 2 citations. The interventional publications were most frequently cited with 2016. The median value was 13 citations. The imaging studies achieved a cumulative total of 567 with a median of 7.

### 3.4 Cooperation map of countries

**Figure 11:**
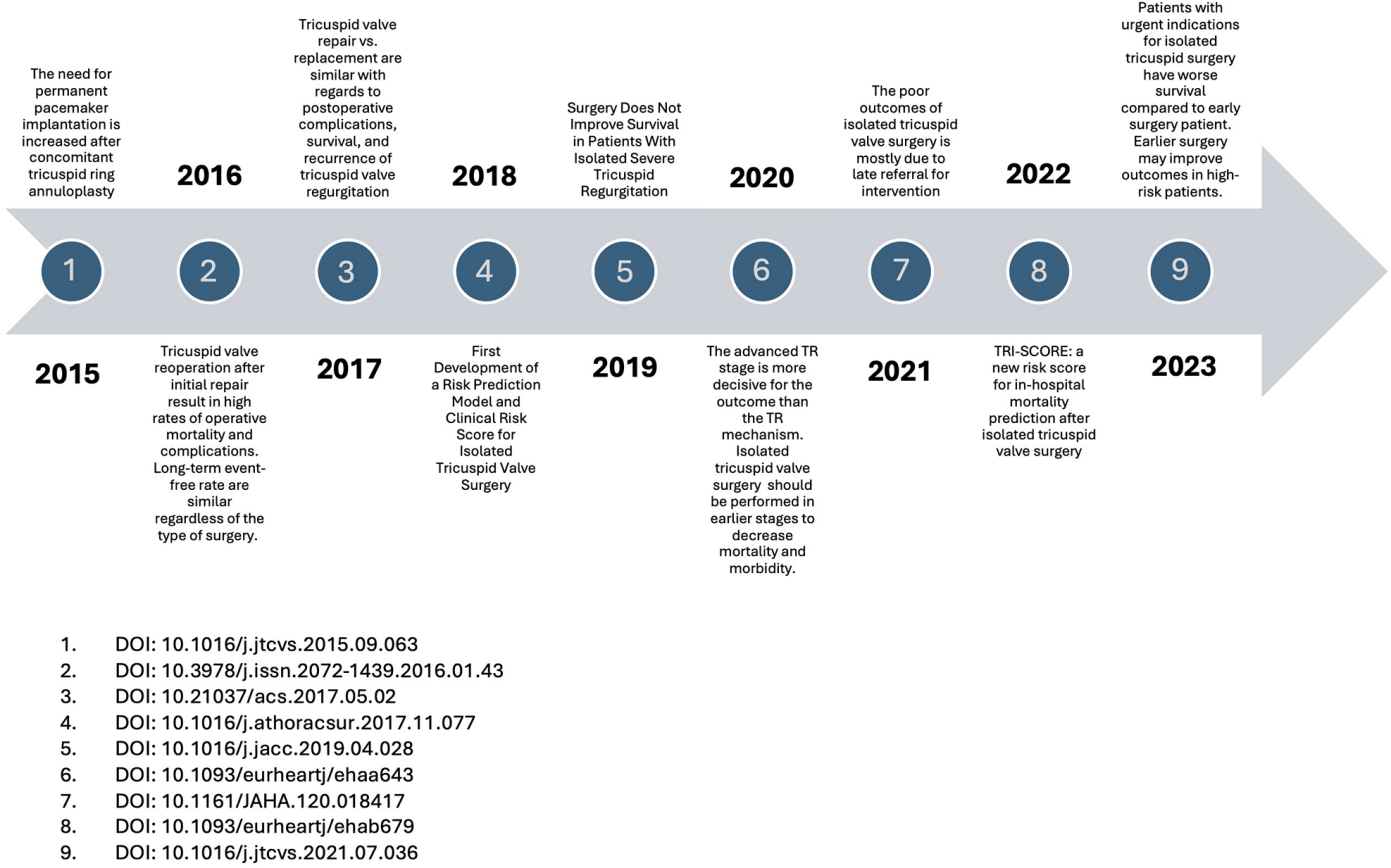
Surgical milestones.

**Figure 12:**
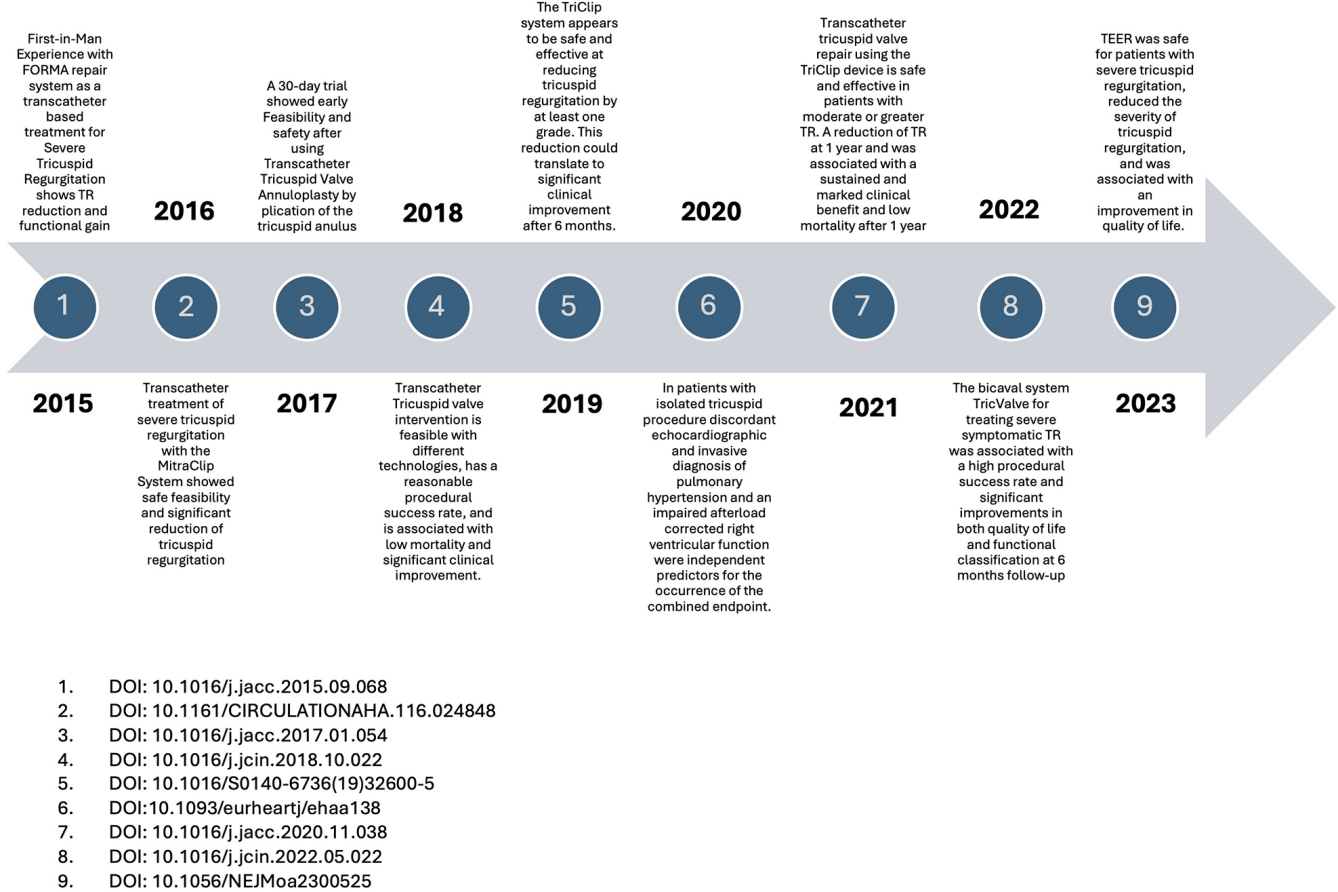
Interventional milestones.

**Figure 13:**
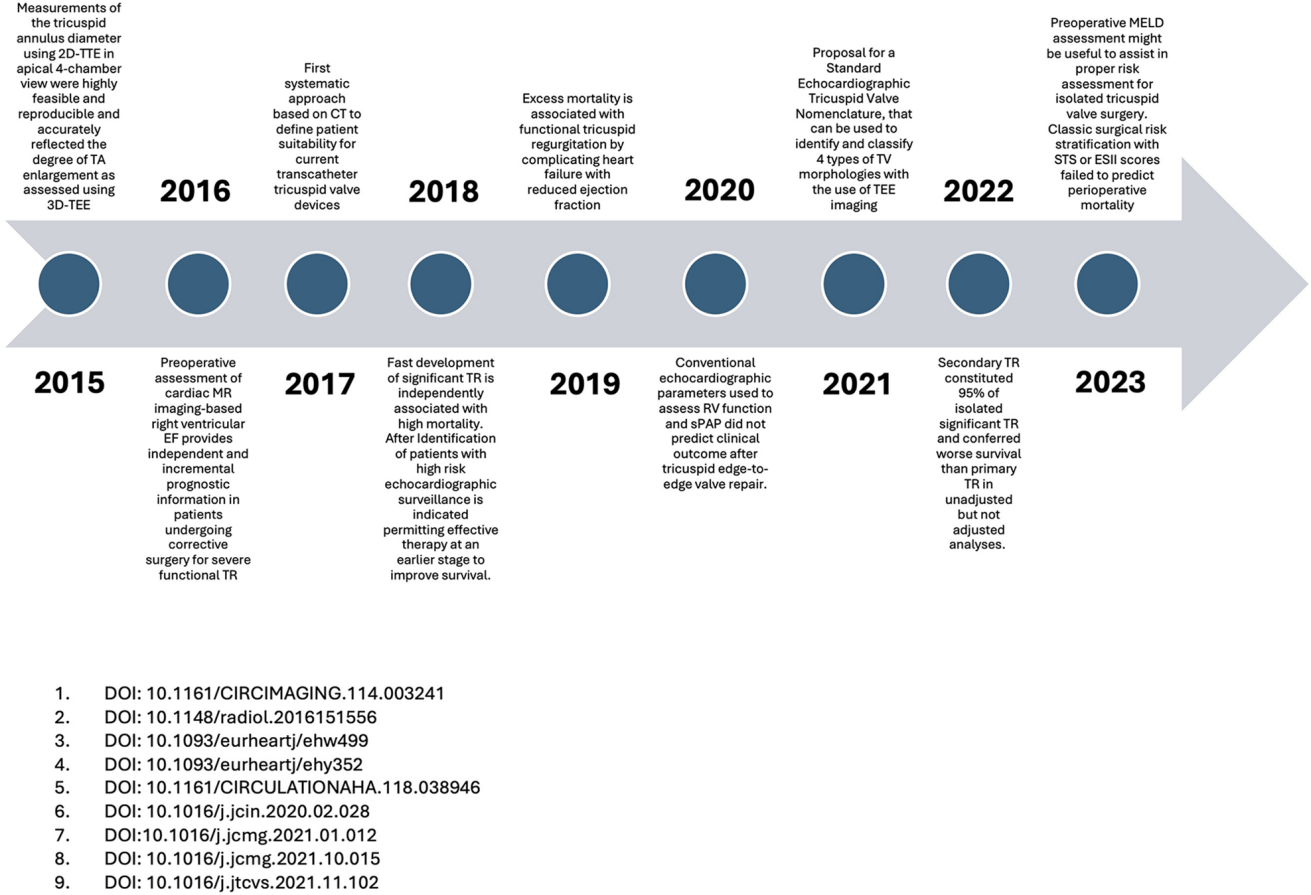
Imaging milestones.

Figures 11–13 show graphic representations of the milestones for the subareas surgical (Figure 11), interventional (Figure 12) and imaging (Figure 13).

### 3.5 Milestones

In the first years of our observation period, the frequently cited surgical publications primarily examined the differences in outcomes between isolated tricuspid valve operations and combined multi-valve operations. The earlier vs. later timing of surgery was also the subject of frequently cited publications. In the further course, valve replacement was compared with valve reconstruction. In more recent surgical research, studies on risk stratification and the introduction of risk scores were cited most frequently. In the most recent top-cited studies, the early timing of surgery is discussed as favourable. In the field of interventional research, first descriptions of new minimally invasive treatment options make up the majority of the most frequently cited publications each year.

Imaging is becoming increasingly important in this area of research, as can be seen from the number of citations over the course of the study period. The first top-cited studies dealt with the comparison between CT-guided, ultrasound- or MRI-guided imaging. With increasing catheter-based interventional options in recent years, many first-in-men studies, especially ultrasound studies, are the most frequently cited.

### 3.6 Analysis of research hotspots

The First-in-Man Experience of a Novel Transcatheter Repair System for Treating Severe Tricuspid Regurgitation was made in 2015 by the Heart & Lung Institute in Quebec, Canada. However, as early as 2016, several universities in Germany emerged as the driving force of interventional innovation with a large number of publications with high impact factor and citation numbers. The universities of Mainz, Bonn, Berlin and the Heart Center Leipzig were significantly involved in the further development and introduction of catheter based tricuspid valve interventions.

Imaging studies have been conducted in many different countries, but the USA has been identified as the leading research nation. In particular, the new classification of tricuspid valve insufficiency based on echocardiographic parameters by the Columbia University research group led by Professor Hahn represents a milestone in imaging research.

In the preclinical studies, the Medical University of Silesia in Katowice and the University of Rzeszow were particularly influential as leading centers with primarily experimental studies.

## 4 Discussion

To the best of our knowledge, this is the first bibliometric study that reflects the development and increase in knowledge in the field of imaging and treatment of TR. The main findings of our study can be summarised as follows:

(1) There has been an enormous increase in knowledge worldwide during 2015–2023, both in quantitative and qualitative terms. (2) In the last 2 years in particular, there has been a similar increase in imaging and interventional publications, whereas the number of primary cardiac surgery studies has decreased drastically. (3) Preclinical studies currently account for the smallest share of total scientific output but have at least remained at a constant level.

After leading a shadowy existence for many years alongside other valvular diseases such as aortic valve stenosis and mitral regurgitation, TR has increasingly become the focus of scientific publications in the last decade. The field developed from only 19 publications in 2015 to a remarkable 69 publications in 2023. This increase, almost by a factor of 3.5, is continuous in all 3 clinical categories apart from a caesura of varying degree in 2019–2022.We suspect a correlation with the Covid pandemic, which could potentially have a significant impact on the focus of global scientific output.

We consider it interesting and remarkable that the publications on imaging and interventional treatment have not increased in parallel in recent years but have tended to increase in the same direction. We clearly attribute this to the fact that interventional treatment is highly dependent on optimal peri-interventional imaging. Vice versa, a broader application of interventional treatments inevitably leads to a further increase in imaging studies in the postinterventional setting.

Germany's high number of publications in this field can be partly attributed to its Diagnosis Related Group (DRG) system, which is highly performance-oriented. Hospitals in Germany generate revenue predominantly through procedures and interventions, which has led to a well-established infrastructure, including a large number of catheterization labs. This system incentivizes interventions as the primary source of income, encouraging a high volume of related research and publications. However, it's important to note that the hard evidence supporting tricuspid valve interventions (TI) is still relatively thin. There is no strong proof yet that these interventions lead to better outcomes in terms of mortality and rehospitalization compared to the simple prescription of diuretics. As a result, in countries with different healthcare priorities and structures, these interventions have a much lower perceived value and importance. Over time, increasing bureaucracy and regulatory demands may be shifting the focus, making it more challenging to continue this high rate of intervention-driven research.

Less easy to explain and interpret is the fact that the number of cardiac surgery publications has decreased in recent years. We know from other valvular diseases such as aortic stenosis that conventional surgery has become less frequent over the years due to interventional treatment options. At this point, however, we would like to emphasise that none of the available interventional treatment options has shown anywhere near the resounding success of transcatheter aortic valve replacement. It should also be noted that the caesura in the years of corona pandemic was most pronounced in the cardiac surgery segment.

This could be due to the lack of intensive care capacities during that time. As a result, scheduled operations without emergency indications could be performed much less frequently. It should also be kept in mind that patients with severe TR often suffer from major comorbidities of other organ systems, so that selection may favour the supposedly gentler interventional treatment. Ultimately, the future will tell whether cardiac surgery treatment is merely lagging or whether interventional treatment is actually superior. In any case, large-scale prospective studies are required to clarify this issue.

The preclinical studies were significantly less present, albeit at a constant level over the years. It is noteworthy that most preclinical publications did not come from the USA, India or China. Within Europe, we were able to identify Germany and Poland as leading countries. The tricuspid valve has historically been considered the “forgotten valve,” as isolated tricuspid valve disease is relatively rare. Tricuspid regurgitation (TR) often occurs as a secondary finding, frequently associated with mitral regurgitation, left ventricular dysfunction (HFrEF), or pulmonary hypertension, which has resulted in a lack of focus on the tricuspid valve itself in both clinical and preclinical research (24). For many years, TR was considered as a byproduct of left-sided heart disease rather than a standalone condition, leading to limited specific studies, especially preclinical ones. The lack of focus has resulted in minimal funding and research into the underlying mechanisms, with few groundbreaking preclinical ideas being explored. Interestingly, the rise of transcatheter tricuspid valve interventions has revealed that the “tricuspid valve” is tricuspid in only 50% of cases. In the remaining cases, it has additional cusps, a fact that has been underappreciated and under-researched until recently **(**25). This multicuspid morphology opens up new areas of investigation, particularly in the areas of pathogenesis, genetics, and embryology, which are still not entirely understood. Moreover, outcomes in patients with multicuspid tricuspid valves are harder to predict, underscoring the need for larger studies with more diverse populations to fully understand the long-term prognosis and optimal treatments for these patients. Further research and dedicated funding are essential to advance our understanding of this complex valve system and its unique challenges.

Unfortunately, there are still major gaps in knowledge about which patient should be assigned to which treatment option. Whether patients are too progressed to benefit from treatment and what the decisive determining factors are. There is still a lack of practicable clinical tools such as biomarkers or clinical scores. We currently have little knowledge about the actual influence of age, gender, liver and kidney disease. As this is a comparatively new scientific field and, in our opinion, there are still considerable gaps in knowledge, we assume that we will not have reached the peak for a long time yet. Further global research activities, both preclinical and comprehensive prospective clinical studies, will be required in order to be able to treat our patients in the best possible way, to estimate prognoses and to clarify the question of allocation, which patient should receive which therapy option.

## 5 Conclusion

TR imaging and its treatment have become the focus of interest over the last 9 years, as evidenced by the significant increase in research on this topic. The USA has emerged as the leading research nation for imaging and Germany for interventions. The research trend is moving away from surgical studies towards interventional studies with accompanying imaging.

## 6 Limitations

We were only able to include studies that could be found using the search algorithm we described. Therefore, it cannot be completely ruled out that there may be other studies in the field. Nevertheless, we are confident that, in consideration of 3,519 screened studies by several independent investigators, the conclusions we have drawn can be considered valid. Using the number of citations as an indicator of the relevance of a publication might have certain issues. It must be borne in mind that newly published studies are not cited as frequently as older ones and might still be of high relevance. As we only used the most frequently cited study each year for our milestones and did not compare individual papers from different years, the significance of the milestones is unrestricted.

A potential limitation of our study is the choice of MeSH terms, particularly the use of “T-TEER” to reflect the growing role of interventional approaches in the treatment of tricuspid regurgitation (TR). While this was intended to capture the full spectrum of therapeutic strategies, including surgical interventions, there is a possibility that some surgical studies may be slightly underrepresented.

## Data Availability

The original contributions presented in the study are included in the article/Supplementary Material, further inquiries can be directed to the corresponding author.
