# Supplementary figures and images for: Bibliometric analysis of imaging and treatment strategies for severe tricuspid regurgitation from 2015 to 2023
Source: Front Cardiovasc Med. 2024 Oct 29;11:1444466. doi: 10.3389/fcvm.2024.1444466 (PMC11554479; doi:10.3389/fcvm.2024.1444466)

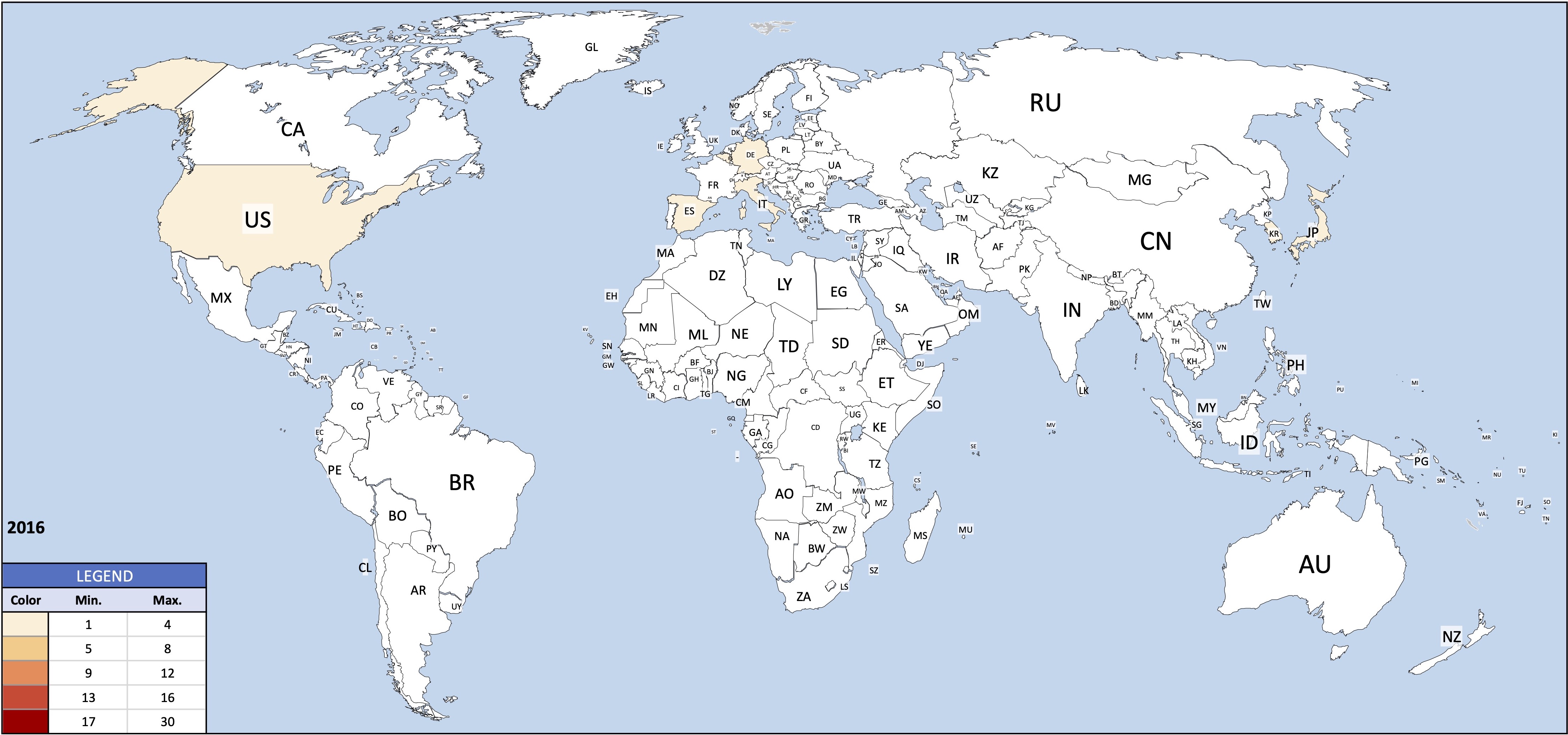

Supplement: Supplementary file 1 [file Image1.jpeg]

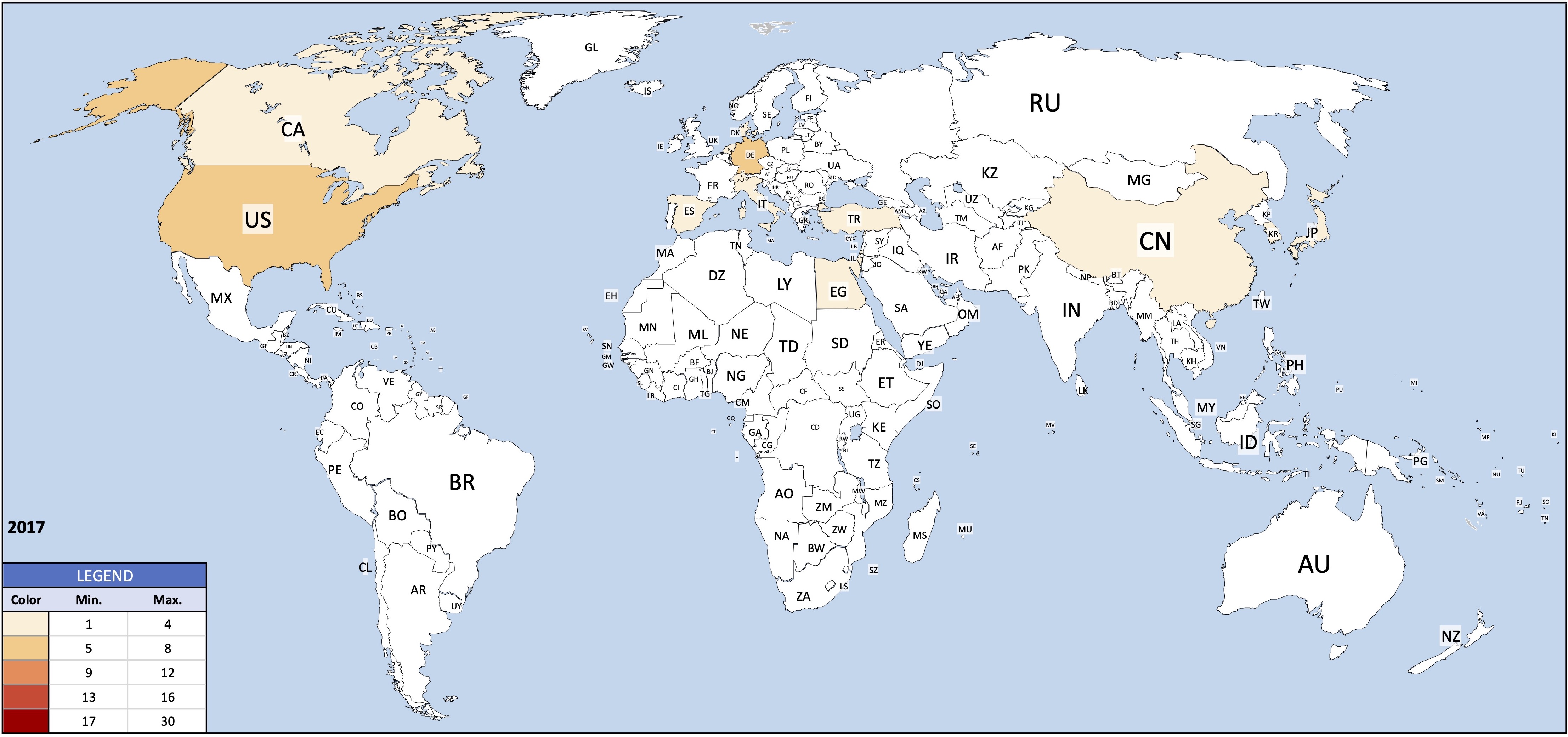

Supplement: Supplementary file 2 [file Image2.jpeg]

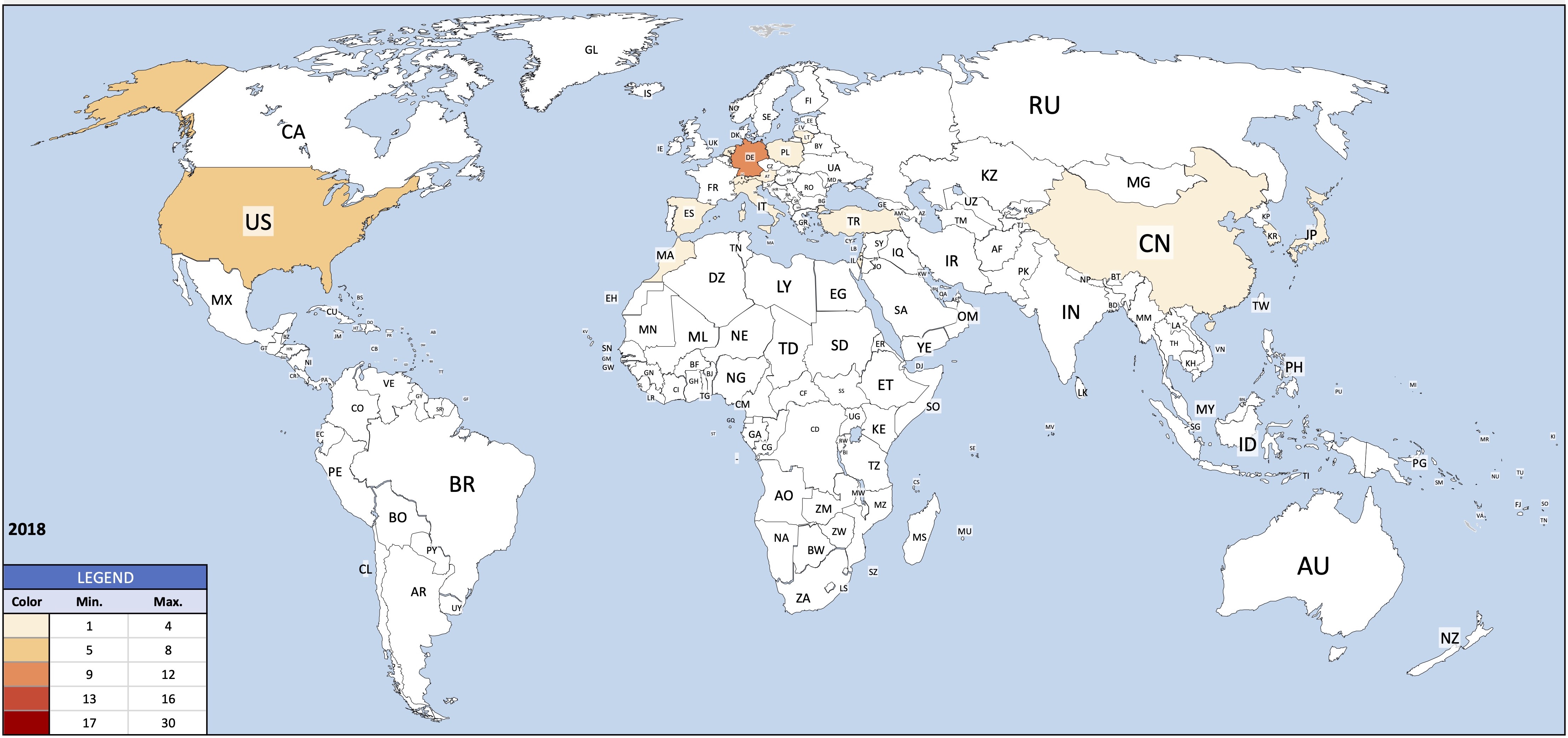

Supplement: Supplementary file 3 [file Image3.jpeg]

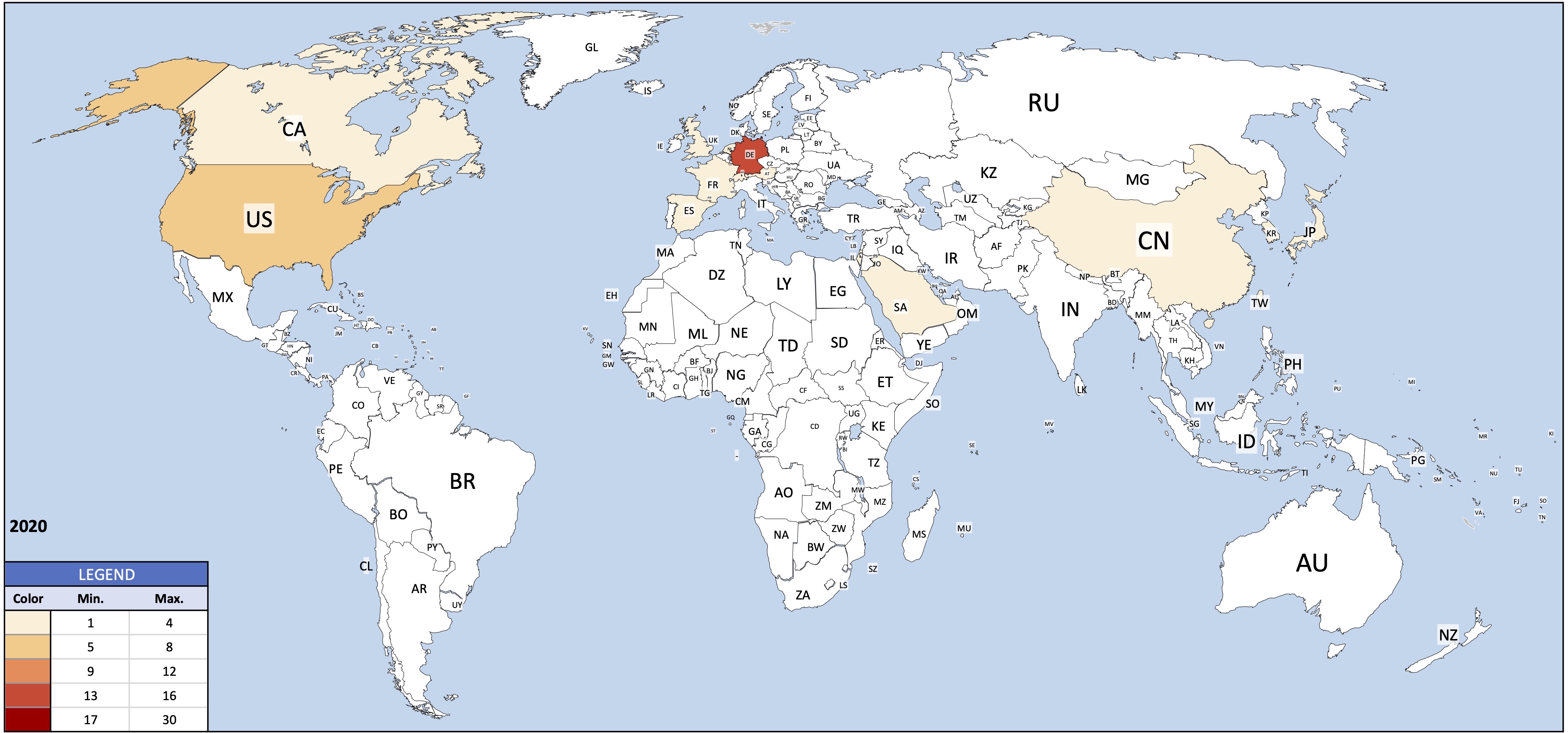

Supplement: Supplementary file 4 [file Image4.jpeg]

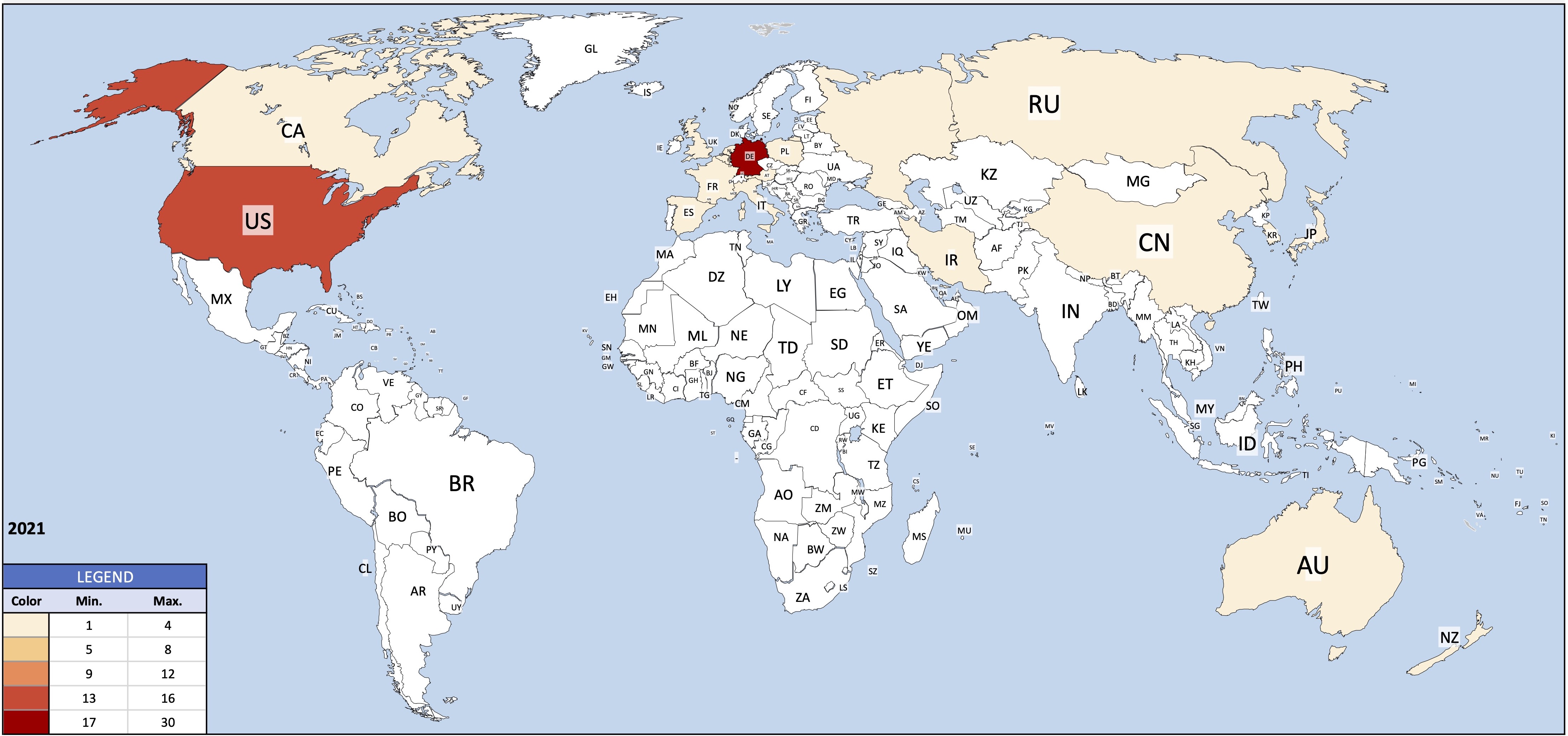

Supplement: Supplementary file 5 [file Image5.jpeg]

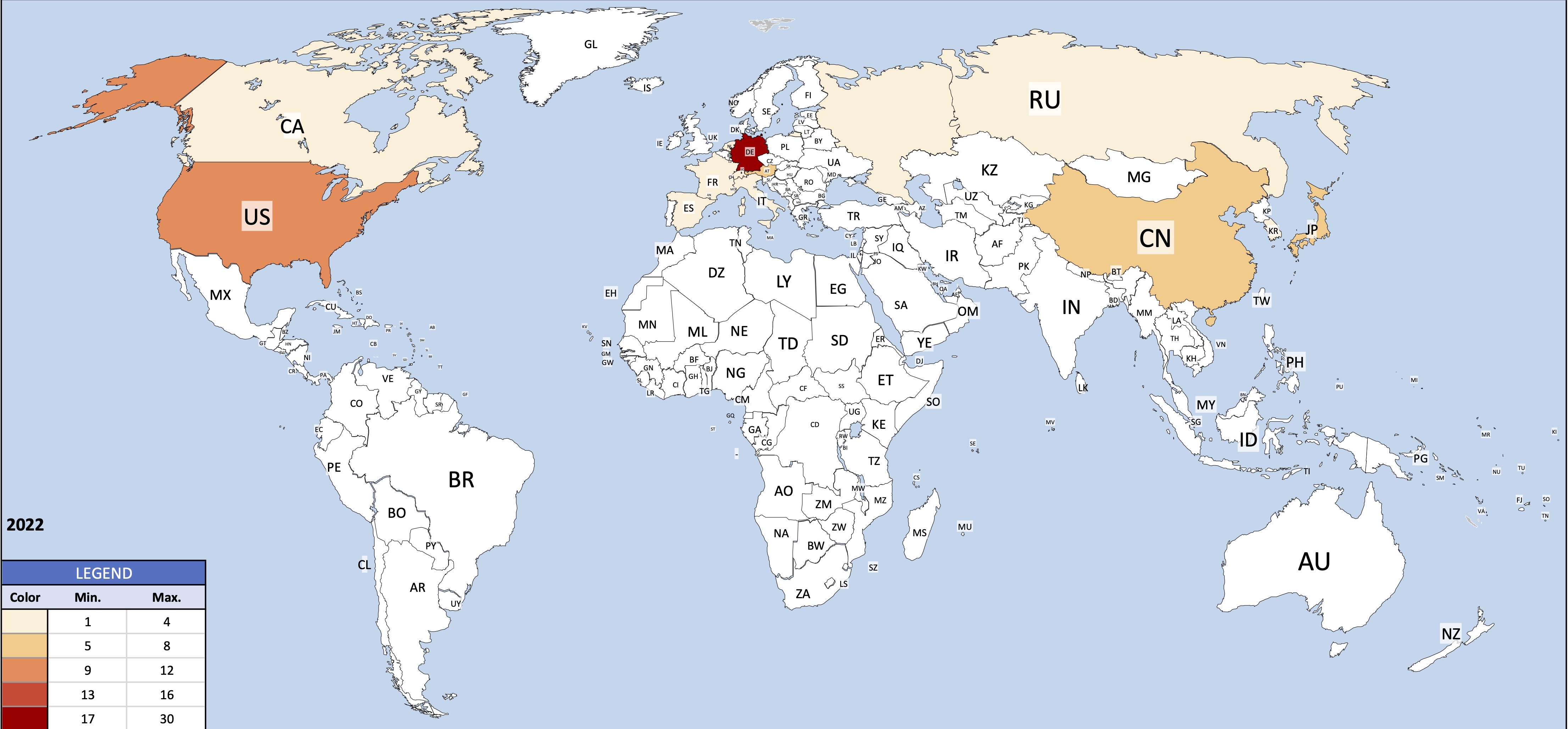

Supplement: Supplementary file 6 [file Image6.jpeg]
